# Bilateral Coronal Synostosis and Mega Cisterna Magna: A Case Report

**DOI:** 10.7759/cureus.25717

**Published:** 2022-06-07

**Authors:** Craig Ballard, Jonathan Deck, Joe Iwanaga, Aaron S Dumont, R. Shane Tubbs

**Affiliations:** 1 Department of Neurosurgery, Tulane University School of Medicine, New Orleans, USA; 2 Neurosurgery, Ochsner Neuroscience Institute, Ochsner Health System, New Orleans, USA; 3 Department of Anatomical Sciences, St. George's University, St. George's, GRD

**Keywords:** anatomy, intracranial, brain, skull, cranium

## Abstract

Craniosynostosis is often associated with raised intracranial pressure (ICP), especially when multiple sutures are involved. In this report, we discuss an unusual association in a patient with craniosynostosis. We report a case of a two-year-old Caucasian male with bilateral coronal synostosis (BCS) who was found to have a concomitant mega cisterna magna (MCM). Although counterintuitive, even in the presence of craniosynostosis, patients with this finding can also have intracranial CSF fluid collections such as the MCM reported here. We hope this report will enhance our understanding of some similar cases that are equivocal regarding raised ICP.

## Introduction

Craniosynostosis, the premature closure of cranial sutures, is a common birth defect and is the second most common craniofacial anomaly after orofacial clefts [1]. This congenital defect can be classified into isolated (nonsyndromic) synostosis, or syndromic synostosis, which is linked to a known heritable gene variant and is often associated with other craniofacial anomalies [2]. Nonsyndromic synostosis accounts for 80-85% of all craniosynostoses [3]. These nonsyndromic synostoses typically involve the premature closure of a single suture [1]. While multiple-suture synostosis is typically associated with a syndrome, the recognition of possibly nonsyndromic multiple-suture synostosis is a recent phenomenon [1]. Retrospective studies have revealed that bilateral coronal synostosis (BCS) is the most common presentation of nonsyndromic multiple-suture synostosis [4,5]. The bilateral coronal sutures are also the most commonly affected in syndromic synostoses [6].

Virchow’s law describes how the fusion of cranial sutures causes compensatory growth along the non-fused sutures, resulting in a predictable pattern of skull deformity [7]. BCS presents with brachycephaly which is described as a shortened skull in the anteroposterior plane with a high forehead [2]. This known pattern allows diagnosis through physical examination of the patient’s skull. The abnormal morphology of the skull puts patients at risk of cerebrospinal fluid (CSF) flow disturbance and increased intracranial pressure (ICP), which has been associated with multiple brain developmental anomalies [1,2,6,8-10].

Due to the wide heterogeneity of craniosynostoses, its treatment requires early diagnosis, surgical correction of the skull deformity within the first year of life, and long-term management of complications involving a multi-disciplinary team [6]. Newly reported phenotypes and associated clinical findings in the literature continually contribute to the collective record of different phenotypes, each with its own unique implications for treatment. We present a case of a patient with BCS and mega cisterna magna (MCM). To our knowledge, there are no other reports of this combination of cranial anomalies in the literature.

This study was conducted according to the requirements of the Declaration of Helsinki (64th WMA General Assembly, Fortaleza, Brazil, October 2013).

## Case presentation

**Figure 1:**
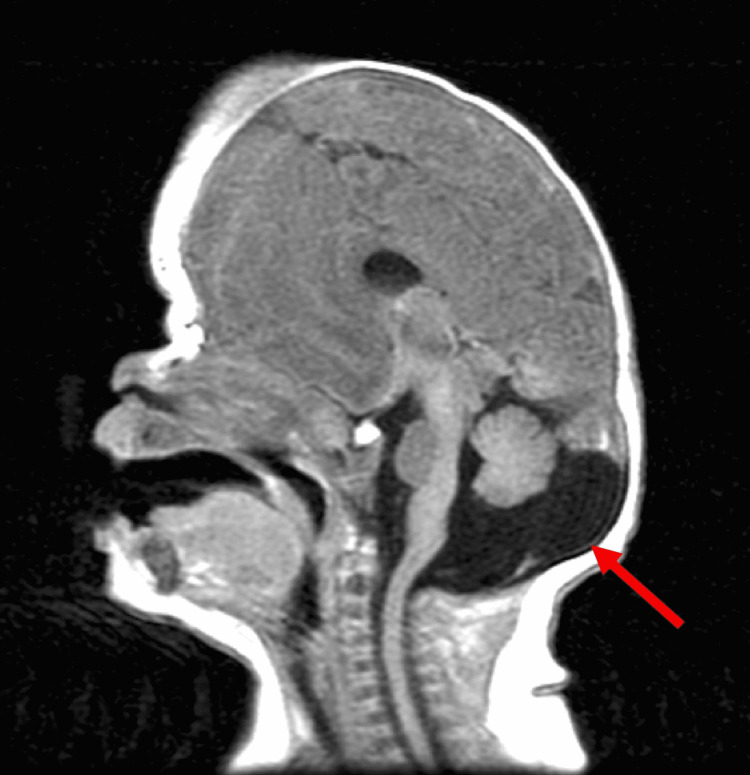
T1-weighted sagittal MRI of the patient. Note the deformation of the skull due to the bilateral coronal synostosis and mega cisterna magna (arrow) MRI: magnetic resonance imaging

A two-year-old Caucasian male presented to our clinic following a referral for an abnormal head shape. He was developmentally normal and the mother reported no symptoms. He was the first child of his mother, the product of 37 weeks of gestation, and had been delivered vaginally. No chromosomal abnormalities or syndromes were identified in this patient. The head circumference was at the 70th percentile for the age. On physical examination, the anterior fontanelle was close and two prominent ridges were palpated anteriorly consistent with the left and right coronal sutures. No midline or frontal ridging consistent with sagittal and metopic synostosis, respectively, were palpated. There was no plagiocephaly. The child moved all extremities and had brisk reflexes throughout. There were no cutaneous stigmata, and the fundoscopic exam was within normal limits. His pupils were symmetrical and reactive, and he followed objects presented to him. An MRI was performed, which revealed no hydrocephalus or intracranial masses, normal patterns of myelination for age, synostosis of the left and right coronal sutures, and a small cerebellum with MCM (Figure 1).

The lambdoid sutures were not synostotic. The remaining intracranial anatomy was felt to be normal, and so was the upper cervical spine.

## Discussion

We presented a case of BCS with MCM, which is a malformation of the posterior cranial fossa [11,12]. This malformation has been linked to several syndromes involving the cerebellum, most notably, the Dandy-Walker spectrum, but is thought to be benign as an isolated finding [13]. To our knowledge, there are no other reports in the literature of BCS in isolation presenting with MCM.

Craniosynostosis can be a part of various syndromes such as Apert’s syndrome, Gomez-Lopez-Hernandez syndrome, and Fontaine-Farriaux syndrome [14,15]. There have also been reports of these syndromes' involvement with cysts such as an MCM [14,15]. Apert’s syndrome can be associated with omphalocele, syndactyly of the hands and feet, cryptorchidism, bicornuate uterus, polycystic kidneys, pulmonary atresia, and MCM [16]. Gomez-Lopez-Hernandez syndrome can be associated with rhombencephalosynapsis, trigeminal anesthesia, and bilateral parietal or parieto-occipital alopecia [17,18]. It has also been associated with cerebellar hypoplasia [17]. Fontaine-Farriaux syndrome is also associated with craniosynostosis. In addition, reports of syndactyly, cryptorchidism, and cerebellar hypoplasia are found in the literature [18]. Our case was not determined to have any of these previously mentioned syndromes.

Central nervous system malformations resulting from mutations/deletions of centrosomal protein 135 (CEP135), T-box brain transcription factor 1 (TBR1), and human zinc finger protein of cerebellum 1 (ZIC1) all contribute to syndromes with craniosynostosis as a common birth defect. TBR1 encodes a transcription factor important in corticogenesis [19]. The transcription factor binds to DNA and regulates the transcription of genes into mRNA leading to the development of the olfactory bulb. Human ZIC1 loss in mice causes cerebellar hypoplasia and vertebral defects. It has a role in cranial suture development by regulating engrailed 1 (En 1). En 1 is a homeobox gene that helps regulate development in the dorsal midbrain and anterior hindbrain (cerebellum and colliculi) of humans. It is also essential in regulating the establishment of a dorsoventral pattern in developing limbs. Gain-of-function mutations in ZlC1 are associated with coronal craniosynostosis and learning disability. It is inherited as an autosomal recessive trait resulting in cerebellar agenesis or cerebellar hypoplasia.

## Conclusions

Counterintuitively, as raised ICP is often found in patients with craniosynostosis, especially with multiple-suture involvement, our case had no signs or symptoms indicative of raised ICP. Furthermore, the presence of an MCM indicated that the ICP was normal. Thus, this case presents a conundrum with regard to the relationship between BCS and MCM. We believe this report might improve our understanding of some similar cases that are equivocal regarding raised ICP.
